# ﻿A new genus and two new species of Opsiini leafhoppers (Hemiptera, Cicadellidae, Deltocephalinae) from China, with a key to genera

**DOI:** 10.3897/zookeys.1228.127783

**Published:** 2025-02-13

**Authors:** Shangmi Hu, Wenjun Cao, Jichun Xing

**Affiliations:** 1 Institute of Entomology, The Provincial Special Key Laboratory for Development and Utilization of Insect Resources, Guizhou University, Guiyang, 550025, China Guizhou University Guiyang China

**Keywords:** Distribution, Homoptera, morphology, new taxa, taxonomy

## Abstract

A new genus of the tribe Opsiini (Hemiptera, Cicadellidae, Deltocephalinae), *Odonaellus***gen. nov.** and two new species, *O.serratus***sp. nov.** (type species) and *O.expansus***sp. nov.**, are described and illustrated; they are placed in the subtribe Eremophlepsiina. A key to subtribes and genera of Chinese Opsiini is provided. The type specimens of the new species are deposited in the Institute of Entomology, Guizhou University, Guiyang, China (GUGC).

## ﻿Introduction

The leafhopper tribe Opsiini belongs to the subfamily Deltocephalinae (Hemiptera, Cicadellidae) with *Opsius* Fieber, 1866 as its type genus. Until now, 42 genera and 353 species were known worldwide. The tribe is identified by the bifurcate aedeagus with two shafts and gonopores (Zahniser and Dietrich 2013). Sixty species belonging to 13 genera have been found in China (Cao and Xing 2022).

In this paper, a new genus *Odonaellus* gen. nov. and two new species from China are described and illustrated: *O.serratus* sp. nov. (type species; Yunnan) and *O.expansus* sp. nov. (Guangxi). Keys to Chinese genera of Opsiini and species of *Odonaellus* are provided. The type specimens of the new species are deposited in the Institute of Entomology, Guizhou University, Guiyang, China (GUGC).

## ﻿Material and methods

Specimens used in this study were collected from Guangxi and Yunnan, China using a sweep net. Dry male specimens were used for the descriptions and illustrations. External morphology was observed under a stereoscopic microscope and characters were measured with an ocular micrometer. A Nikon SMZ1270 microscope was used to dissect the male genitalia. Color images for adult habitus and male genitalia were obtained using the Keyence VHX-6000 system. The genital segments of the examined specimen were macerated in 10% NaOH. Images were imported into Adobe Photoshop CS8 for labeling and plate composition. Morphological terminology follows Li et al. (2011).

## ﻿Taxonomy

**Tribe Opsiini Emeljanov, 1962**

### ﻿Key to subtribes and genera of Opsiini from China

Modified from Dai et al. 2010; Cao and Xing 2022.

**Table:** 

1	Subgenital plates with macrosetae well developed and conspicuous	**2**
–	Subgenital plates with macrosetae absent or greatly reduced (**Eremophlepsiina**)	**3**
2	Mesal margin of eye notched, single T-branched shaft arising from base of aedeagus, with shaft branches forming semicircle (**Circuliferina**)	***Neoaliturus***
–	Mesal margin of eye not notched, aedeagal shafts arising from base separately (**Opsiina**)	**4**
3	Aedeagus shafts denticulate mesally on distal half	***Odonaellus* gen. nov.**
–	Aedeagus shafts without denticulation	***Pseudophlepsius***
4	Subgenital plate with an additional lateral plate at base	***Alishania***
–	Subgenital plate without additional lateral plate	**5**
5	Pygofer with paired sharp lateral process arising ventrally	***Norva***
–	Pygofer without process	**6**
6	Crown, pronotum, scutellum and forewings with a few, or no, brown spots	**7**
–	Crown, pronotum, scutellum and forewings with numerous scattered brown spots	**9**
7	Aedeagal socle swollen and bulbous	***Opsius***
–	Aedeagal socle not swollen	**8**
8	Aedeagal shafts with a subapical process	***Japananus***
–	Aedeagal shafts with three or four subapical processes	***Japananoides***
9	Forewings with a triangular marble pattern forming in the middle of a darker rhomboidal spot when wings at rest	***Hishimonus***
–	Forewings without triangular marble pattern forming in the middle of a darker rhomboidal spot when wings at rest	**10**
10	Aedeagus with atrium extending ventrad of shafts	***Litura***
–	Aedeagus with atrium not extending ventrad of shaft	**11**
11	Aedeagus without basal process arising from socle	**12**
–	Aedeagus with basal process arising from socle	**13**
12	Forewings with symmetrical longitudinal orange stripes	***Yinformibus***
–	Forewings without symmetrical longitudinal orange stripes	***Orosius***
13	Aedeagus with two or three pairs of shafts	***Hishimonoides***
–	Aedeagus with pair of shafts divided near base	***Introrsa***

### ﻿Subtribe Eremophlepsiina Dmitriev, 2002

#### ﻿ Odonaellus gen. nov.

Taxon classificationAnimalia

EDF1D0E0-46E3-5ABF-A2AE-BBDF7E1B3082

https://zoobank.org/CDE39C57-F5A6-46C8-8556-424FB53F99E9

##### Type species.

*Odonaellusserratus* sp. nov.

##### Description.

Body yellowish-brown. Crown yellow, with white stripe and a pair of small black spots at apex of crown connected to a black transverse marginal band. Eyes dark brown; ocelli yellowish-brown and on anterior margin of head. Pronotum with anterior yellowish brown and posterior brown. Face pale brown. Forewing yellowish, with brown arched lines and patchy spots. Legs brownish yellow.

Body robust. Head including eyes narrower than pronotum; crown slightly produced medially, shorter than width between eyes; ocelli on anterior margin, separated from corresponding eye by approximately their own diameter; face slightly flattened, its width narrower than length; anteclypeus slightly expanded apically. Pronotum obviously longer than wide, anterior margin strongly and roundly produced, posterior margin slightly concave. Scutellum triangular, wider than long, slightly longer than pronotum, with transverse suture depressed. Forewing hyaline, about 3 times as long as wide, with 4 apical cells and 3 subapical cells; inner subapical cell closed. Fore femur with 2 dorsoapical setae; row IC with stout setae; row AV with short, stout setae; tibia with 2 rows of setae. Hind femur broadened distally and slightly bowed; apical setal formula 2+2+1; tibia flattened and nearly straight, row PD with 28 macrosetae decreasing in length toward base; row AD with approximately 13 long stout setae and 0–4 shorter stout setae between each long seta.

***Male genitalia*.** Male pygofer slightly longer than high, with paired ventral process or dorsal process, without macrosetae, and setae on posterior margin. Valve subtriangular. Subgenital plate without macrosetae or with a few small macrosetae and with wide base, slightly narrowed posteriorly, without digitiform apical extension. Aedeagal shaft arising from base with paired shafts, denticular, curved, U-shaped in ventral view, gonopore subapical. Connective Y-shaped, articulated with aedeagus. Style broad at base, subapically slightly concaved.

##### Remarks.

This new genus is placed in subtribe Eremophlepsiina based on the following: crown concavely depressed, with a pair of apical black submedial maculae; head narrower than the pronotum; wings macropterous; macrosetae on the male subgenital plate reduced or absent, pygofer with paired posterior processes, aedeagus with shafts arising from the base; and the female ovipositor extending far beyond the pygofer apex. The genus is distinguished from other Eremophlepsiina by the lack of irregular brown markings on the head and pronotum, the much less prominent brown vermiculate markings on the forewing, and the apically denticulate shafts of the aedeagus. The two included species differ for some characters mentioned in the subtribal diagnosis provided by Zahniser and Dietrich (2013), suggesting that the genus may be intermediate between Eremophlepsiina and Opsiina (see Remarks for individual species below).

##### Etymology.

The new genus name is derived from the Latin word “*odona*” and the diminutive suffix “-*ellus*”, in reference to the denticulate (tooth-like) processes on the aedeagal shaft. Gender: masculine.

##### Distribution.

Oriental region (China).

### ﻿Key to species of *Odonaellus* gen. nov. from China (males)

**Table:** 

1	Pygofer process arising from posterodorsal part of lobe (Fig. 5); aedeagus with base as long as shafts in ventral view (Fig. 10)	***O.serratus* sp. nov.**
–	Pygofer process arising from posteroventral part of lobe (Fig. 21); aedeagus with base much shorter than shafts in ventral view (Fig. 26)	***O.expansus* sp. nov.**

#### ﻿ Odonaellus serratus sp. nov.

Taxon classificationAnimalia

6C7905DC-524C-5486-91AE-0D701E7A5893

https://zoobank.org/F7F35958-C834-4091-8158-DD8F222FCE02

Figs 1–4 , 5–12

##### Description.

**Figures 1–4.**
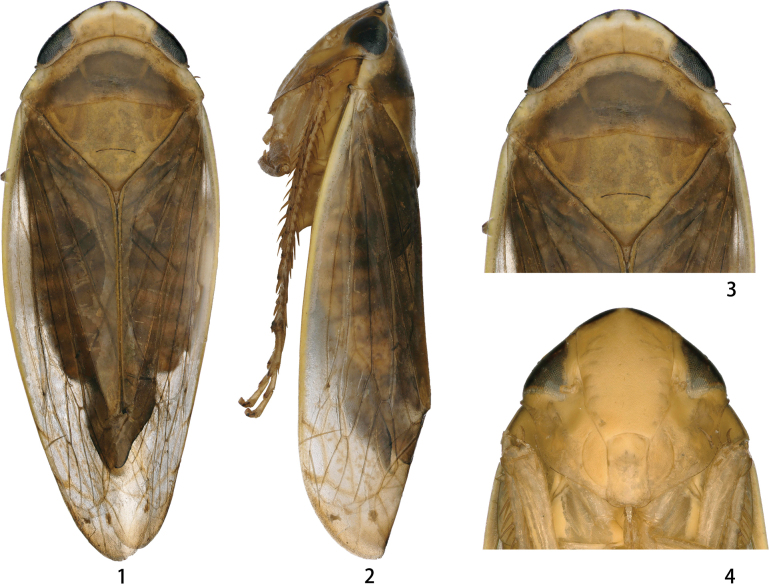
*Odonaellusserratus* sp. nov. **1** ♂, dorsal view **2** ♂, lateral view **3** ♂, head and thorax, dorsal view **4** ♂, face.

Color pattern of anterior dorsum and face as in Figs 1–4. Face yellow with a few brown stripes (Fig. 4).

##### Other external features as in generic description.

**Figures 5–12.**
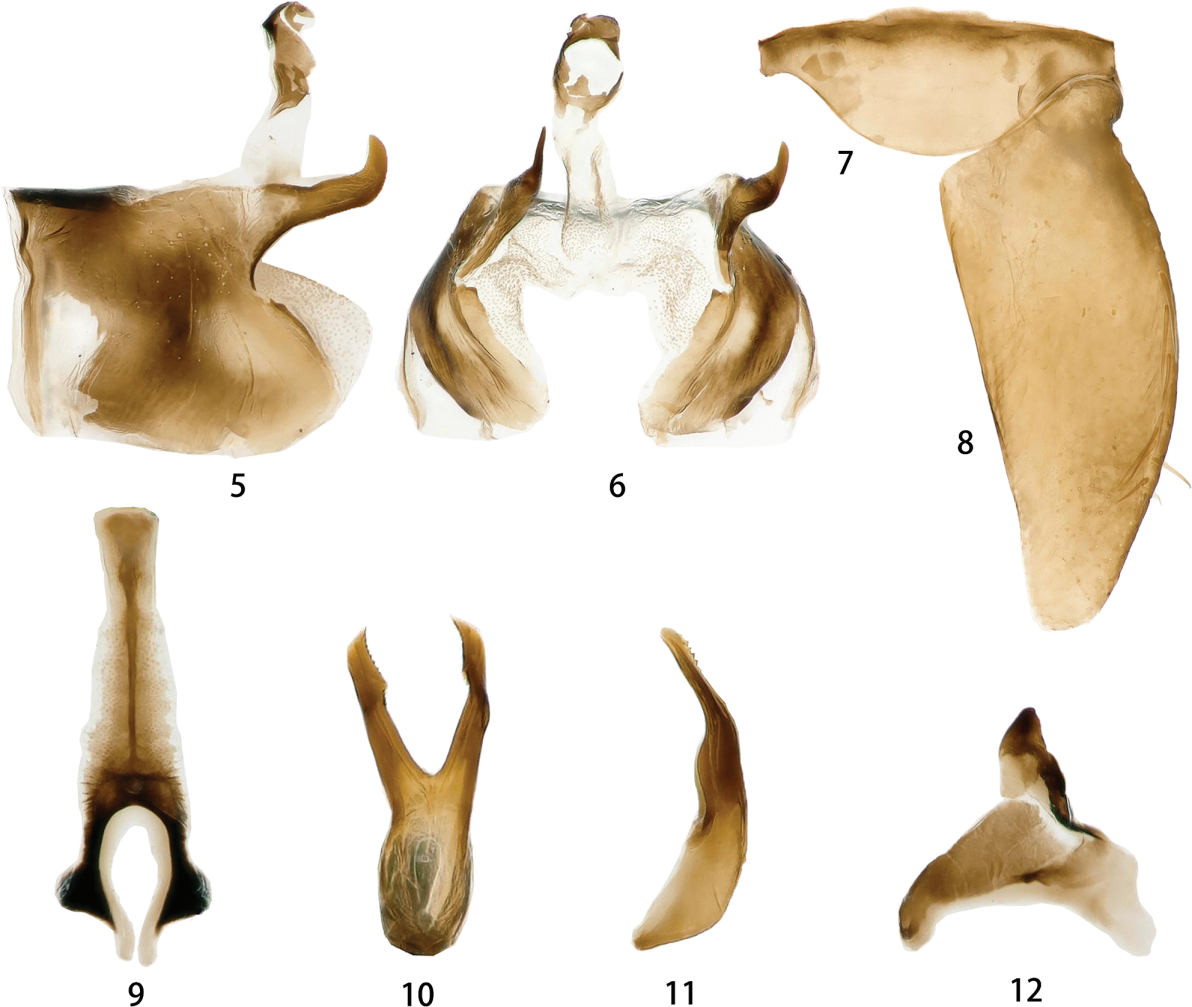
*Odonaellusserratus* sp. nov. **5** male pygofer side, lateral view **6** male pygofer side, ventral view **7** valve, ventral view **8** subgenital plates, ventral view **9** connective, ventral view **10** aedeagus, ventral view **11** aedeagus, lateral view **12** style, dorsal view.

***Male genitalia*.** Male pygofer with one pair of ﬁnger-like dorsal processes arising caudally and with a few macrosetae along caudal margin; ventral margin expanded (Figs 5, 6). Valve small (Fig. 7). Subgenital plate with a few stout setae (Fig. 8). Aedeagal shaft lamellar, apical tapered and apcial inner margin dentate; gonopore subapical (Figs 10, 11). Connective with arms shorter than stem (Fig. 9). Style curved, apical apophysis stout and subapically concaved (Fig. 12).

***Measurement*.** Length (including tegmen): ♂, 5.3–5.7 mm.

##### Type material.

***Holotype***: • ♂, China: Yunnan, Mengla County, Mohan; 21°22'30"N, 101°75'46"E; 13 May 2015, coll. Qiang Luo (GUGC); ***paratypes***: • 5 ♂♂, Yunnan, Mengla County, Mohan; 21°13'21"N, 101°44'59"E; 13 May 2015, coll. Qiang Luo; • 3 ♂♂, Yunnan, Mengla County, Menglun; 21°91'32"N, 101°26'83"E; 12 May 2015, coll. Qiang Luo (GUGC).

##### Etymology.

The new species name is Latin adjective *serratus*, “serrate”, used in reference to the apically aedeagal shaft toothed like a saw.

##### Remarks.

This species disagrees with the subtribal diagnosis of Eremophlepsiina, as provided by Zahniser and Dietrich (2013), in having small macrosetae present laterally on the male subgenital plate, the pygofer process arising dorsally rather than ventrally, and the valve distinctly shorter than wide.

#### ﻿ Odonaellus expansus sp. nov.

Taxon classificationAnimalia

56F9F325-A87E-5CB6-8056-46FFBFA5438F

https://zoobank.org/662E89F3-915E-44F4-A0C3-3746755C2528

Figs 13–16 , 17–20 , 21–28 , 29–34

##### Description.

Color pattern of anterior dorsum and face as in Figs 13–20. Male pronotum dark brown with irregular fuscous spots (Fig. 15); female pronotum yellowish brown, without spot (Fig. 19).

##### Other external features as in generic description.

***Male genitalia*.** Male pygofer with one pair of sharp ventral processes arising caudally and a pair of tiny posteroventral process grown in dorsal inner surface (Figs 21, 22). Valve large (Fig. 23). Subgenital plate without macrosetae (Fig. 24). Aedeagal shaft auricular and subapical inner margin with a few small dentae; gonopore subapical (Figs 26, 27). Connective with arms shorter than stem (Fig. 25). Style curved; apical apophysis stout and subapically concave. (Fig. 28).

**Figures 13–16.**
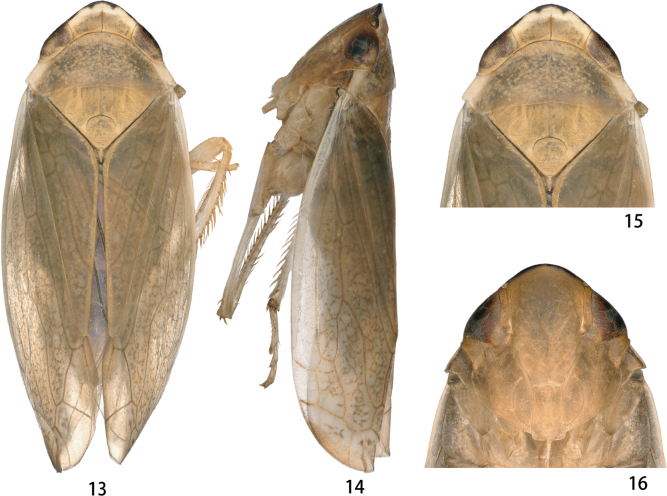
*Odonaellusexpansus* sp. nov. **13** ♂, dorsal view **14** ♂, lateral view **15** ♂, head and thorax, dorsal view **16** ♂, face.

**Figures 17–20.**
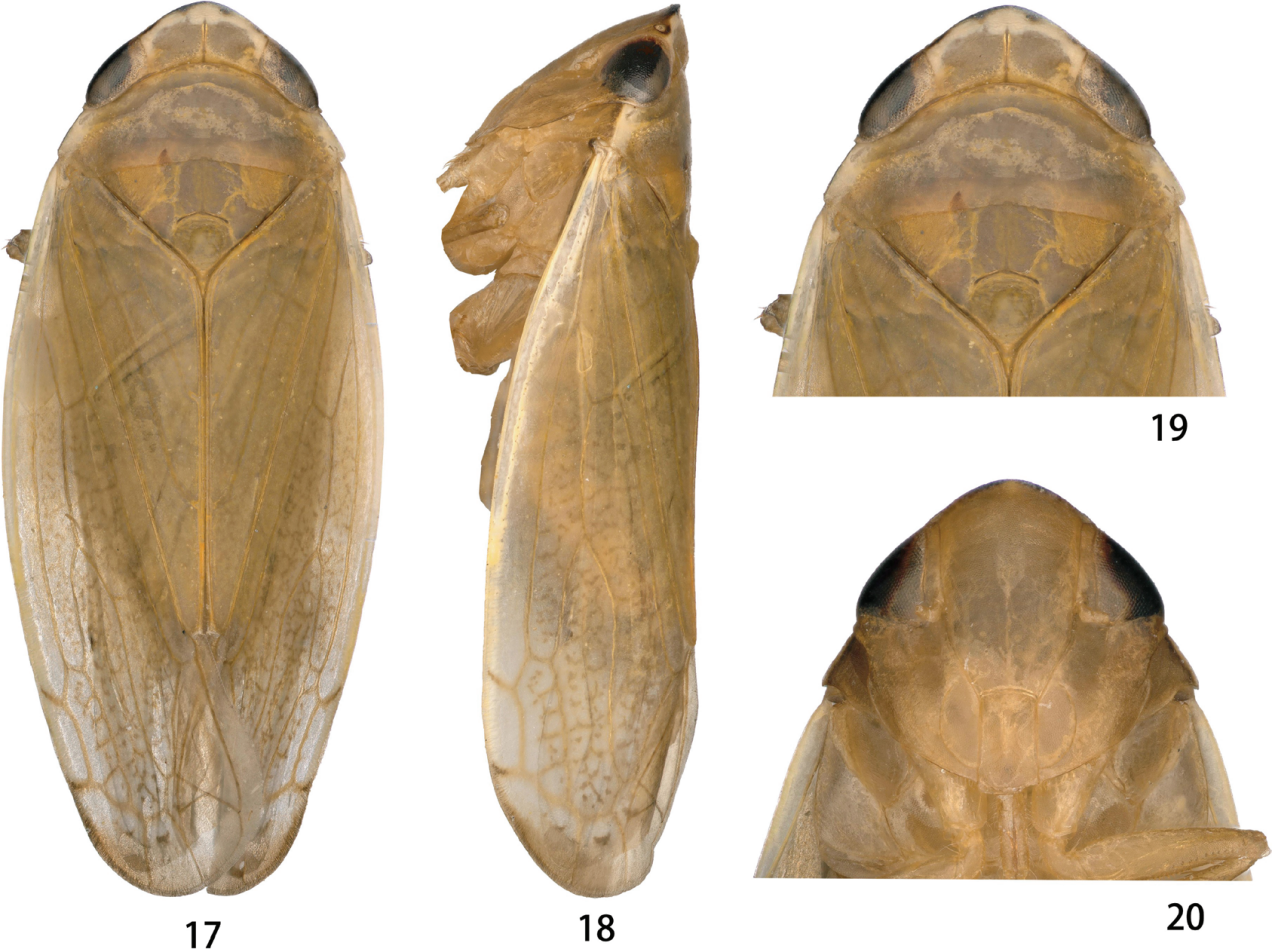
*Odonaellusexpansus* sp. nov. **17** ♀, dorsal view **18** ♀, lateral view **19** ♀, head and thorax, dorsal view **20** ♀, face.

**Figures 21–28.**
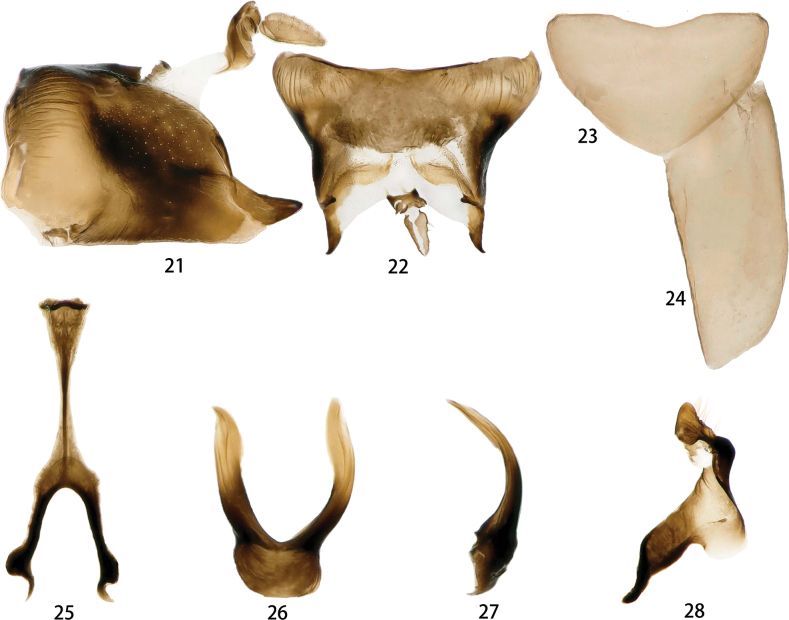
*Odonaellus* sp. nov. **21** male pygofer side, lateral view **22** male pygofer side, dorsal view **23** valve, ventral view **24** subgenital plates, ventral view **25** aedeagus, ventral view **26** aedeagus, lateral view **27** connective, ventral view **28** style, dorsal view.

**Figures 29–34.**
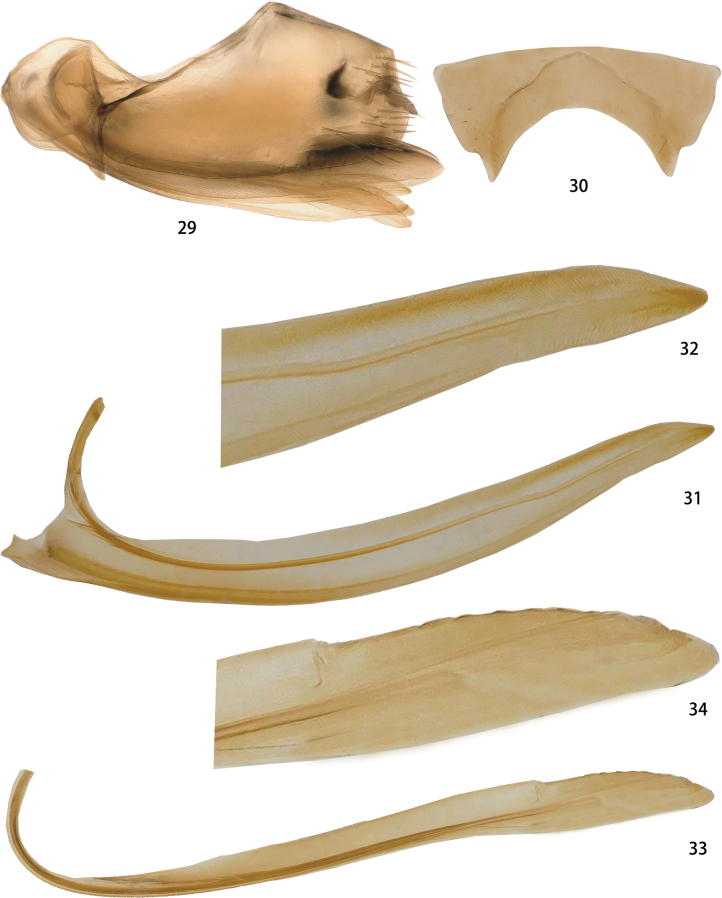
*Odonaellusexpansus* sp. nov. **29** female genital capsule, lateral view **30** seventh sternite, ventral view **31** first valvula, lateral view **32** detail of sculptures of first valvula **33** second valvula, lateral view **34** detail of sculpture on second valvula.

Female pygofer with ventroposterior margin sharply incurved (Fig. 29). Female seventh sternum posterior margin concave, resulting in projection on both sides (Fig. 30). First valvula of ovipositor slightly curved, attenuate to apex, with indistinct scale-like sculpture ventrally (Figs 31, 32). Second valvula with small teeth near apex (Figs 33, 34).

***Measurement*.** Length (including tegmen): ♂, 5.4–5.7 mm; ♀, 5.8–6.5 mm.

##### Type material.

***Holotype***: • ♂, China: Guangxi Autonomous Region, Longzhou County, Nonggang; 22°25'21"N, 106°97'27"E; 4 May 2014, coll. Qu Wu (GUGC); ***paratypes***: • 4 ♂♂ 5 ♀♀, Guangxi Autonomous Region, Longzhou County, Nonggang; 22°49'97"N, 106°97'49"E; 8 May 2012, coll. Zhiwei Fan, Hu Li; • 3 ♂♂, Guangxi Autonomous Region, Longzhou County, Nonggang; 22°52'66"N, 106°96'94"E; 4 May 2014, coll. Qu Wu (GUGC).

##### Etymology.

The new species name is Latin adjective *expansus*, “expanded”, which is in reference to the broadly expanded base of the aedeagal shaft.

##### Remarks.

This species agrees more closely with the subtribal diagnosis of Eremophlepsiina (Zahniser and Dietrich 2013) than does the type species of the genus. Unlike *O.serratus*, *O.expansus* has the male valve nearly as long as wide, the pygofer process ventrally arising, and the subgenital plate lacking macrosetae as in other Eremophlepsiina.

## Supplementary Material

XML Treatment for Odonaellus

XML Treatment for Odonaellus serratus

XML Treatment for Odonaellus expansus

## ﻿Additional information

### Author ORCIDs

Shangmi Hu https://orcid.org/0009-0007-3824-5993

Wenjun Cao https://orcid.org/0000-0002-1865-2598

Jichun Xing https://orcid.org/0000-0002-3738-7996

### Data availability

All of the data that support the findings of this study are available in the main text.
